# Microbial solutions must be deployed against climate catastrophe

**DOI:** 10.1038/s41522-024-00591-9

**Published:** 2024-11-11

**Authors:** Raquel Peixoto, Christian R. Voolstra, Lisa Y. Stein, Philip Hugenholtz, Joana Falcao Salles, Shady A. Amin, Max Häggblom, Ann Gregory, Thulani P. Makhalanyane, Fengping Wang, Nadège Adoukè Agbodjato, Yinzhao Wang, Nianzhi Jiao, Jay T. Lennon, Antonio Ventosa, Patrik M. Bavoil, Virginia Miller, Jack A. Gilbert

**Affiliations:** 1 International Society for Microbial Ecology (ISME), Arnhem, the Netherlands; 2 International Coral Reef Society (ICRS), Tavernier, FL USA; 3 King Abdullah University of Science and Technology, Thuwal, Saudi Arabia; 4 Department of Biology, University of Konstanz, Konstanz, Germany; 5 University of Alberta, Edmonton, Alberta, Canada; 6 University of Queensland, Brisbane, Queensland, Australia; 7 University of Groningen, Groningen, the Netherlands; 8 New York University Abu Dhabi, Abu Dhabi, United Arab Emirates; 9 Federation of European Microbiological Societies (FEMS), Cambridge, UK; 10 Rutgers University, New Brunswick, NJ USA; 11 University of Calgary, Calgary, Alberta, Canada; 12 Stellenbosch University, Stellenbosch, South Africa; 13 Shanghai Jiao Tong University, Shanghai, China; 14 Université d’Abomey-Calavi UAC, Abomey Calavi, Benin; 15 Global Ocean Negative Carbon Emissions (ONCE) Program, Research Center for Ocean Negative Carbon Emissions, Fujian, China; 16 Xiamen University, Fujian, China; 17 American Society for Microbiology (ASM), Washington DC, USA; 18 American Academy of Microbiology (AAM), Washington DC, USA; 19 Indiana University, Bloomington, IN USA; 20 University of Sevilla, Seville, Spain; 21 University of Maryland, College Park, MD USA; 22 University of North Carolina at Chapel Hill, Chapel Hill, NC USA; 23 Applied Microbiology International (AMI), Cambridge, UK; 24 University of California San Diego, La Jolla, CA USA

**Keywords:** Water microbiology, Microbial ecology

## Abstract

This paper is a call to action. By publishing concurrently across journals like an emergency bulletin, we are not merely making a plea for awareness about climate change. Instead, we are demanding immediate, tangible steps that harness the power of microbiology and the expertise of researchers and policymakers to safeguard the planet for future generations.

The climate crisis is escalating. A multitude of microbe-based solutions have been proposed (Table 1), and these technologies hold great promise and could be deployed along with other climate mitigation strategies. However, these solutions have not been deployed effectively at scale. To reverse this inaction, collaborators across different sectors are needed — from industry, funders and policymakers — to coordinate their widespread deployment with the goal of avoiding climate catastrophe. This collective call from joint scientific societies, institutions, editors and publishers, requests that the global community and governments take immediate and decisive emergency action, while also proposing a clear and effective framework for deploying these solutions at scale.

**Table 1:** Examples of microbial strategies that can be developed and/or deployed at scale to tackle climate change^1–3,10^

Strategy	Mechanism of action	Benefits	Application
Carbon sequestration	Microbial enhancement of carbon sequestration in soils and oceans	Reduces atmospheric CO_2_ and enhances soil productivity	Agricultural and forestry sustainability and marine biosequestration
Methane oxidation	Use of methanotrophic bacteria to oxidize methane into less harmful compounds	Lowers methane emissions and can promote atmospheric removal; mitigates a potent greenhouse gas	Landfills; livestock management; inland freshwater bodies; wetlands
Bioenergy production	Cultivation of algae and other microbes for biofuel production	Provides renewable energy; reduces reliance on fossil fuels	Biofuel production; industrial applications
Bioremediation	Microbial breakdown of pollutants and hazardous substances	Improves environmental health; reduces toxin exposure	Industrial waste management; contaminated land and sediment restoration
Microbial therapies	Targeted microbiome management using microbial therapies (for example, probiotics, postbiotics, prebiotics); can mitigate harmful microbiomes and consequent environmental degradation; restoring beneficial microbiomes across hosts and ecosystems	Improves organismal and environmental health and can be applied to sustainable practices, which, in turn, minimizes greenhouse gas emissions	Wildlife and ecosystem restoration and rehabilitation; sustainable agriculture; human health
Nitrogen management	Engineering crops with symbiotic bacteria to fix atmospheric nitrogen or crops that produce biological nitrification inhibitors	Enhances soil fertility; reduces fertilizer use; increases plant nitrogen use efficiency; decreases eutrophication and greenhouse gas emissions	Sustainable agriculture; crop production

## Microbes and the climate crisis

**Fig. 1:**
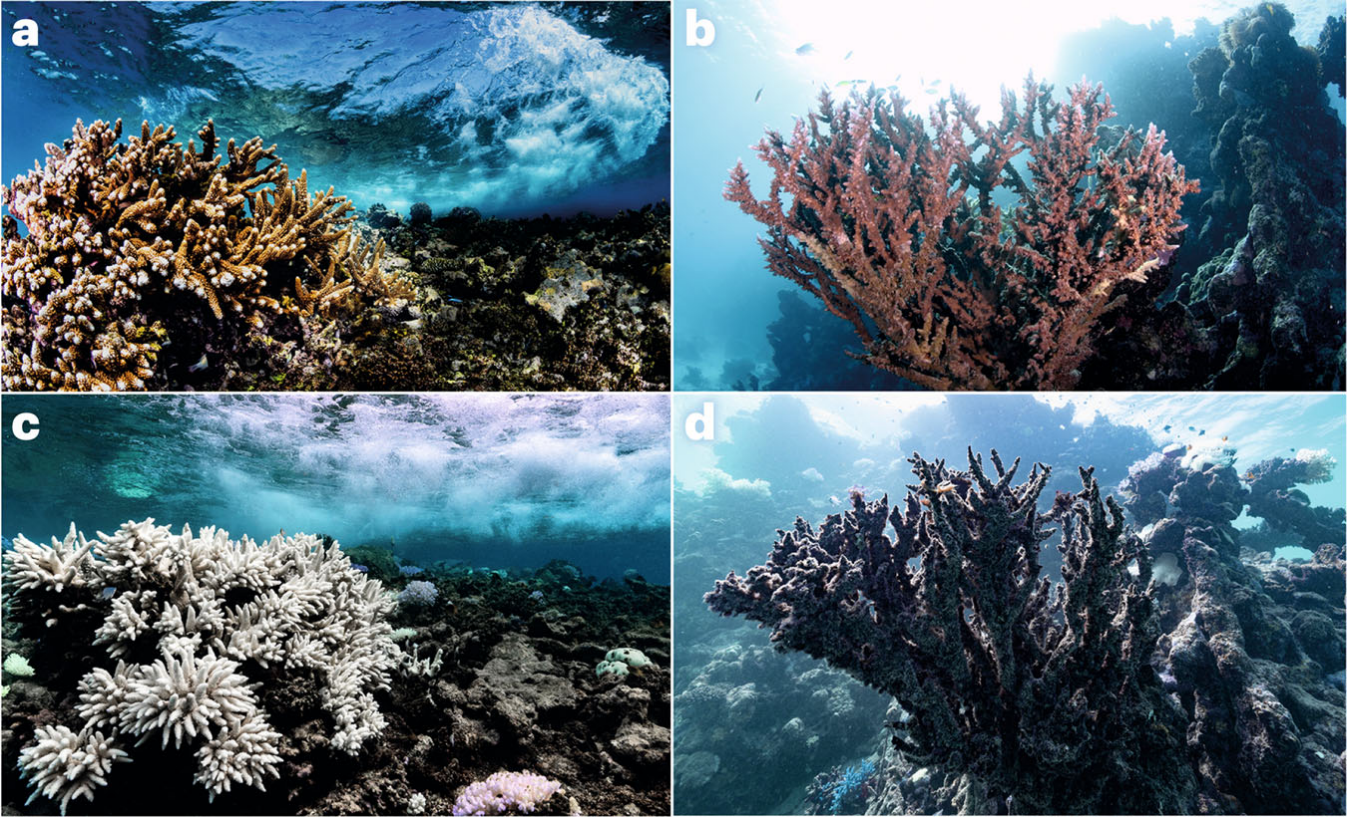
Corals and climate change. **a**–**d** Examples of the same healthy (**a,** **b**), bleached (**c**) and dead (**d**) corals before (**a,** **b**) and after (**c, d**) being affected by heatwaves caused by climate change. Photos by Morgan Bennett-Smith.

Microorganisms have a pivotal but often overlooked role in the climate system^1–3^ — they drive the biogeochemical cycles of our planet, are responsible for the emission, capture and transformation of greenhouse gases, and control the fate of carbon in terrestrial and aquatic ecosystems. From humans to corals, most organisms rely on a microbiome that assists with nutrient acquisition, defence against pathogens and other functions. Climate change can shift this host–microbiome relationship from beneficial to harmful^4^. For example, ongoing global coral bleaching events, where symbiotic host–microbiome relationships are replaced by dysbiotic (that is, pathogenic) interactions (Fig. 1), and the consequent mass mortality mean the extinction of these ‘rainforests of the sea’ may be witnessed in this lifetime^5^. Specifically, a decline of 70–90% in coral reefs is expected with a global temperature rise of 1.5 °C (ref. ^6^). Although this example highlights how the microbiome is inextricably linked to climate problems, there is a wealth of evidence that microbes and the microbiome have untapped potential as viable climate solutions (Table 1). However, despite the promise of these approaches, they have yet to be embraced or deployed at scale in a safe and coordinated way that integrates the necessary but also feasible risk assessment and ethical considerations^7^.

## Mobilizing microbiome solutions to climate change

The multifaceted impacts of climate change on the environment, health and global economy demand a similar, if not more urgent and broad, mobilization of technologies as observed in response to the COVID-19 pandemic^8,9^. To facilitate the use of microbiome-based approaches and drawing from lessons learned during the COVID-19 pandemic^9^, we advocate for a decentralized yet globally coordinated strategy that cuts through bureaucratic red tape and considers local cultural and societal regulations, culture, expertise and needs. We are ready to work across sectors to deploy microbiome technologies at scale in the field.

We also propose that a global science-based climate task force comprising representatives from scientific societies and institutions should be formed to facilitate the deployment of these microbiome technologies. We volunteer ourselves to spearhead this, but we need your help too. Such a task force would provide stakeholders such as the Intergovernmental Panel on Climate Change (IPCC) committee and United Nations COP conference organizers, and global governments access to rigorous, rapid response solutions. Accompanied by an evidence-based framework, the task force will enable pilot tests to validate and scale up solutions, apply for dedicated funding, facilitate cross-sector collaboration and streamlined regulatory processes while ensuring rigorous safety and risk assessments. The effectiveness of this framework will be evaluated by key performance indicators, assessing the scope and impact of mitigation strategies on carbon reduction, ecosystem restoration and enhancement of resilience in affected communities, aiming to provide a diverse and adaptable response to the urgent climate challenges faced today. We must ensure that science is at the forefront of the global response to the climate crisis.

We encourage all relevant initiatives, governments and stakeholders to reach out to us at climate@isme-microbes.org. We are ready and willing to use our expertise, data, time and support for immediate action.
